# Supplementary material for: Development of a computer-aided design software for the quantitative evaluation of aesthetic damage
Source: PLoS One. 2019 Dec 18;14(12):e0226322. doi: 10.1371/journal.pone.0226322 (PMC6919621; doi:10.1371/journal.pone.0226322)
Supplement: S1 Code site — 1. (ZIP) [file pone.0226322.s001.zip › [EESC_jr][Projeto]Relatorio_07_21/vendors/select2/docs/_includes/nav/announcements-4.0.html]

- Select2 4.0.0
  - New features
  - Plugin system
  - AMD-based build system
- Migrating from Select2 3.5
  - No more hidden input tags
  - Advanced matching of searches
  - More flexible placeholders
  - Display reflects the actual order of the values
  - Changed method and option names
  - Deprecated and removed methods

Back to top
